# The Korean Version of the Academic Cyberincivility Assessment Questionnaire for Nursing Students in South Korea: Validity and Reliability Study

**DOI:** 10.2196/15668

**Published:** 2020-05-05

**Authors:** Minjoo Hong, Jennie C De Gagne, Hyewon Shin, Suhye Kwon, Gum-Hee Choi

**Affiliations:** 1 Department of Nursing Gyeongnam National University of Science and Technology Jinju Republic of Korea; 2 School of Nursing Duke University Durham, NC United States; 3 School of Nursing Clemson University Greenville, SC United States; 4 College of Nursing Kosin University Busan Republic of Korea; 5 Choonhae College of Health Sciences Ulsan Republic of Korea

**Keywords:** cybercivility, health professions education, nursing students, social media, web-based learning

## Abstract

**Background:**

Cybercivility, the practice of what to say and how to say it in online environments, encourages individuals to treat each other with respect. However, the anonymity of online communities may lead some individuals to behave in ways that violate social and cultural norms. These individuals treat others with a lack of regard and even bully others in faceless online confrontations. This practice of cyberincivility can be found across the internet, on commercial sites, and in schools offering online courses. Research on cybercivility and cyberincivility has increased in the United States, where instruments have been developed to measure the impact of cyberincivility in health profession education. However, there is no available instrument that measures nursing students’ online behaviors in South Korea.

**Objective:**

The aim of this study was to develop and evaluate a Korean version of the Academic Cyberincivility Assessment Questionnaire developed in the United States.

**Methods:**

Data were collected from 213 nursing students in three South Korean colleges. The Academic Cyberincivility Assessment Questionnaire developed by De Gagne and colleagues was adapted to measure students’ knowledge of cybercivility, and their experiences with and acceptability of cyberincivility. Content validity was tested using the content validity index (CVI). Criterion validity was tested using the digital citizenship scale. Reliability was evaluated using Cronbach alpha. The goodness-of-fit of construct validity was determined through exploratory and confirmatory factor analyses.

**Results:**

The CVI was 0.8 or higher for all items. Kuder–Richardson Formula 20, measuring reliability of the knowledge scale, was 0.22 and Cronbach alpha, measuring reliability of the experience scale, was .96. The goodness-of-fit of the model was Chi square=5568.63 (*P*<.001), the comparative fit index (CFI) was 0.92, and the root mean square error of approximation (RMSEA) was 0.08, which satisfied the criteria. The reliability of the acceptability scale was .96, and the goodness-of-fit indices satisfied the criteria (minimum Chi square/df=2.34, Tucker-Lewis Index =0.92, incremental fit index=0.93, root mean square residual=0.05, CFI=0.93, and RMSEA=0.08).

**Conclusions:**

This study extended and reevaluated the US version of cybercivility scales in a culturally distinct context. The three dimensions of cybercivility include knowledge, experience, and acceptability. Acceptability is well-validated as a dimension, whereas the knowledge dimension requires reexamination for application to Koreans. A revision of the instrument is needed that considers the cultural differences between South Korea and the United States. This paper calls for more attention to be paid to contextualized cybercivility scales among health professions in countries outside the United States.

## Introduction

### Background

Internet and mobile devices are essential to everyday life in the digital age. Most individuals use the internet and social media to keep in touch with friends and family, locate goods and services, and educate themselves on a variety of topics, including health [1]. In South Korea, more than 95% of the population uses smartphones, and the country’s use of the internet is among the highest in the world [2]. Moreover, South Korea has an advanced health information technology infrastructure [3]. The use of mobile devices as a means for health information dissemination is well documented [2] and makes South Korea a promising market for such interventions in nursing and nursing education.

The expansion of online networks in which information is exchanged through social networking services (SNSs) has increased opportunities for communication, education, and socialization, but has also led to inappropriate online environment activities [4]. Due to the relative anonymity of individuals meeting online, boundaries are skewed and it is difficult to avoid uncivil exchanges [5,6]. Behaviors that cause harm or unpleasantness in online environments are caused by a lack of awareness as to how opinions, memes, and videos might affect others [7]; a lack of civility in communicating and cooperating with others online; or a lack of literacy that causes well-intentioned posts to convey unintended meanings [8,9]. Cybercivility, defined as the practice of respect and responsibility in the online environment [10], makes it possible to exchange new ideas and make new connections, which is especially important in social networking sites and virtual learning environments.

Cyberincivility is an issue that spans across age and professional groups [1,5]. Recently, research on the behavior of nurses and nursing students revealed a substantial lack of cybercivility, including offensive racial and ethnic remarks through a SNS, posting information without evidence as professional knowledge, using profanity, or violating the confidentiality of patients and coworkers [11,12]. Clinicians or practicum students may breach confidentiality due to lack of civility in online communities by posting patient information via SNSs, providing inaccurate medical information, or posting photos that violate professional ethics [13]. Thus, it is important to provide clear guidelines and education for health profession majors, including nursing students. De Gagne et al [14] developed instruments that measure the knowledge, direct/indirect experience, and perception of cybercivility of health profession students. The authors addressed the issue that future health care professionals experience cyberincivility and perceive incivility as a growing problem. Thus, future health care providers need to be educated on ethical and professional standards, including those that apply in online environments. Although a digital citizenship instrument has been developed to measure incivility in South Korea, since this tool concerns political and global issues [15], Cf it is insufficient to examine the cybercivility of nursing students. Therefore, the aim of the present study was to translate the cyberincivility assessment questionnaire developed in the United States [14] from English into Korean and evaluate its psychometric properties.

### Objectives

The aim of this study was to develop a Korean version of the Academic Cyberincivility Assessment Questionnaire (ACAQ) [14] and evaluate its reliability and validity among nursing students in South Korea. These findings could be used as the basis of education about cybercivility for students in health profession education.

## Methods

### Study Design and Participants

We translated the English version of the ACAQ into Korean and tested the psychometrics of the measures with a survey of nursing students from December 2018 to February 2019. The study participants were students at universities in the Busan and Gyeongnam provinces of South Korea. Approximately 14% of Korean nursing departments are located in these two medium-sized cities in urban locations, which are similar with respect to socioeconomic status. Participants were eligible for inclusion in the study if they were: (1) nursing students and (2) aged 18 years and older. Paper questionnaires consisted of a total of 80 items that included 9 questions about demographics, 15 questions about knowledge, and 28 questions each related to experiences and acceptability of cyberincivility. To perform a factor analysis on the data, the sample size needed to be about five times the number of items in the questionnaire [16]. The appropriate sample size needed for a confirmatory factor analysis (CFA) is at least 200 [17]. The sample size for this study was 213, which satisfied the sample size requirement.

### Ethical Considerations

After obtaining approval from the Kosin University Institutional Review Board (KU IRB 2018-0095), we advertised the study on campus. The principal investigator and team members provided paper questionnaires to the researchers or research assistants who explained the research purpose and methods to participants in person. Participation was voluntary, and we explained to the participants that they could drop out at any time during the study. As compensation for their efforts, each participant was given a gift worth the equivalent of 3 USD. We collected 222 surveys, 213 of which were included in the final analysis after excluding 9 surveys due to incomplete information. Permission to use the ACAQ was granted from the original developer of the instrument designed for health profession students (ie, medicine, nursing, physician assistant, and physical therapy) in the United States [14]. Permission from the authors was also obtained for use of the digital citizenship instrument [15] for assessment of criterion validity of the scale.

### Measurements

#### Questionnaire Development

To assess health profession students’ knowledge of cybercivility, and their experience with and perceptions of behaviors related to cyberincivility, De Gagne et al [14] developed the ACAQ. The aim of the present survey was also to determine cybercivility learning needs related to interprofessional education (IPE) core competencies in medical, nursing, physician assistant, and physical therapy programs [14]. The ACAQ consists of 75 items in the following 4 sections: (1) demographics, (2) knowledge about cybercivility, (3) experience and perceptions of cyberincivility, and (4) perceived benefits of including cybercivility education as part of the IPE curriculum. Because the present study surveyed only nursing students and IPE pedagogy is not well-understood among Korean nursing students, we excluded section 4 (perceived benefits of including cybercivility in IPE and preferred formats) from our survey.

#### Demographics

Demographic questions were based on those in the original studies related to cybercivility [9,14]. The items included in the demographics were gender, year in school, clinical practice experience, SNS memberships (multiple responses), time spent on SNS per day, and number of text messages sent per day.

#### Knowledge of Cybercivility

The original ACAQ contains 15 items to test students’ knowledge of uncivil behaviors in online environments [14]. Response choices were “true,” “false,” and “I don’t know,” with a score of 1 and 0 assigned for correct and incorrect answers, respectively; a score of 0 was also assigned for a response of “I don’t know.” The range of calculated scores was 0-15; the higher the score, the higher the knowledge of cybercivility. The original scale of the reliability measured by Kuder-Richardson formula 20 (KR-20) was 0.58. The scores for KR-20 range from 0 to 1, with a score closer to 1 indicating greater reliability of the test. In general, a score above 0.5 is usually considered to be reasonable [18].

#### Experience With and Acceptability of Cyberincivility

The original ACAQ includes 28 items in two areas (ie, experience and acceptability) that are responded on a 5-point Likert scale [14]. Respondents were asked to rate how often they had experienced or observed uncivil events described in the questionnaire (1=*never*, 2=*rarely*, 3=*occasionally*, 4=*frequently*, 5=*very often*) and how acceptable they perceived each behavior to be (1=*not at all acceptable*, 2=*slightly acceptable*, 3=*moderately acceptable*, 4=*very acceptable*, 5=*extremely acceptable*). Participants were also asked to report how frequently they experienced or observed their peers, instructors, and other individuals demonstrating certain behaviors. Acceptability was measured by asking the participants to rank the acceptability of the behavior based on its actual or potential consequence(s) related to the students’ professional or personal development. Cronbach alpha for the original scale on experience and acceptability with cyberincivility was .95 and .94, respectively.

#### Digital Citizenship

The digital citizenship instrument developed by Choi and Park [15] to determine the concept of citizenship in the digital age was used as the criterion for evaluating the validity of experience and acceptability of the cyberincivility instrument. This instrument is composed of 23 items in the following five areas evaluated on a 7-point Likert scale: (1) internet political participation, (2) technical ability to use the internet, (3) critical perspective, (4) online communication and collaboration, and (5) sensitivity to community and global issues. The digital citizenship scale has good reliability and construct validity that is supported by an expert panel review, exploratory factor analysis (EFA), and CFA [19]. The digital citizenship scale has been used in several previous studies [19,20]. To determine the reliability of a scale, testing/retesting is necessary, and it should be confirmed that the scales are similar after a retest by applying the same surveys to the same participants after a certain period [21]. However, retesting the same participants would have been difficult and there was the possibility of a decrease in the accuracy due to the prior measurement for this survey. Therefore, we did not use a test-retest method but rather evaluated the validity using the correlations with an existing digital citizenship scale. In a pilot study, there was no ceiling or floor effect observed [22]. After receiving permission from the author, we converted the digital citizenship scale to a 5-point scale to facilitate comparison with the survey of cybercivility. A higher score on this scale indicates a higher level of agreement about digital citizenship. The overall Cronbach alpha was .81 in the original study and was .89 in this study.

### Procedure

#### Instrument Translation and Back Translation

Translation of the instrument was conducted based on World Health Organization guidelines [23] in the order of: (1) preliminary translation, (2) expert panel, (3) back translation, (4) preliminary survey, (5) determination of criterion-related validity, and (6) completion. A bilingual nursing professor working at a nursing college in the United States translated the original instrument into Korean. The translation was confirmed by an investigator who is fluent in both Korean and English. Each item on the scale was verified with the original author for accuracy. For example, item 8 in the *knowledge of cybercivility* section (“Americans encounter incivility almost equally offline and online”) was revised with consent of the original author because we assumed cultural differences between South Korea and the United States. Thus, the new statement, “People tend to be ruder online than they are in everyday life,” was added to maintain the same number of items in the knowledge section. Similarly, item 14 in the *experience with and perceptions of cyberincivility scale,* “Using displays of attitude such as capitalizing or boldfacing words in an argument,” was also modified to “Using new words or abbreviations that seem to mock the other person in discussions in cyberspace” as the Korean language does not have capitalization or bold text. In the expert panel review, there was a comment on possible unfamiliarity of the terms and concepts of cybercivility or sociocultural differences. Therefore, we reflected on these comments as we translated the instrument. Phrases in the translated instrument were verified by a professor of the Korean language to ensure accuracy. The Korean translation of the experience and acceptability items of the ACAQ is presented in Multimedia Appendix 1.

#### Preliminary Survey

A preliminary survey was given to 24 nursing students from May 2 to 12, 2018 using the newly translated questionnaire. The purpose of the preliminary survey was to determine the clarity of the content, whether the terminology was easily understood, and if the 15 to 20 minutes allotted to complete the survey was reasonable. The students responded that the survey time was sufficient to answer all questions, and that the items were easy to understand.

### Statistical Analysis

The data were analyzed using SPSS 20.0 (IBM Corp, Armonk, NY, USA) and AMOS 22 (IBM SPSS AMOS, Version 22.0. 2013, IBM Corp, Armonk, NY, USA) software. Descriptive statistics, including distribution, were calculated for the demographic variables and item scores. The content validity of the experience and acceptability scales was assessed using the content validity index for individual items (I-CVI) and for scales (S-CVI/Ave). To that end, a 5-member expert panel of four nursing professors and one professor of education scored each item on a 4-point scale (1=not relevant, 2=somewhat relevant, 3=quite relevant, 4=highly relevant).

The I-CVI for each item is computed as the number of experts giving a rating of 3 or 4 divided by the number of experts, and the S-CVI/Ave for the scale is calculated as the mean of the I-CVI values of all items on the scale [21]. The criterion validity of the knowledge section was tested by grouping participants according to whether or not they had taken online classes. To take online classes, students need basic knowledge about and the ability to use the internet [24]; therefore, we assumed that there might be a knowledge gap between those who had taken an online class and those who had not.

The internal consistency and reliability were evaluated using the Cronbach alpha reliability coefficient, which normally ranges between 0 and 1; Cronbach alpha of .8 is considered to be a reasonable goal [25]. The Kaiser-Meyer-Olkin (KMO) measure of sampling adequacy and the Bartlett test of sphericity were conducted to determine the appropriateness of the data for EFA. CFA was used to examine the factor structure. The construct validity was then assessed with EFA using maximum-likelihood estimation with oblique promax rotation. The coefficients for the frequency and acceptability of the cyberincivility items were computed along the eigenvalues of the factors. Each factor was interpreted through examination of the item content, patterns of factor structures, and factor pattern coefficients. Following the EFA, a series of maximum-likelihood estimations via CFA were conducted using AMOS 22.0. The fit of the model was verified by the minimum Chi square/degrees of freedom (CMIN/df) value, comparative fit index (CFI), Tucker-Lewis index (TLI), standardized root mean square residual (SRMR), and root mean square error of approximation (RMSEA). To verify the validity of the criteria, the correlation between experience/acceptability of cyberincivility and digital citizenship was analyzed using Pearson correlation coefficient (*r*), which ranges from –1 to 1; *r* of –1 indicates a perfect negative relationship, *r* of +1 indicates a perfect positive relationship, and *r* of 0 indicates no linear relationship between variables [25]. The strength of the correlation for the absolute value of *r* was interpreted as very week for *r*=0.00-.019, weak for *r*=0.20-0.39, moderate for *r*=0.40-0.59, strong for *r*=0.60-0.79, and very strong for *r*=0.80-1.0 [26].

## Results

### Descriptive Statistics

The basic characteristic of the participants are summarized in Table 1. The majority of the participants were women in their 20s who were third-year nursing students with clinical practice experience. The most frequent number of SNS memberships was 1-5, and the most frequently accessed SNS was KakaoTalk, which is a free smartphone app for messaging that is used by most Korean smartphone owners, followed by Facebook, Instagram, Naver Band, KakaoStory, and Twitter. The majority of respondents had taken online classes. Most of the respondents (128/213, 88.3%) with online class experience indicated that the greatest advantage of asynchronistic online classes was the convenience of fitting classes into their personal and professional schedules, while the most common disadvantage reported (101/213, 68.7%) was limited interactions with their professors and peers.

**Table 1:** Descriptive statistics of survey respondents (N=213).

Characteristic		Value
Age (years), mean (SD)		22.58 (0.78)
**Gender, n (%)**		
	Male	26 (12.2)
	Female	187 (87.8)
**Year in school, n (%)**		
	2^nd^	40 (18.8)
	3^rd^	114 (53.5)
	4^th^	59 (27.7)
**Clinical practice experience, n (%)**		
	Yes	173 (81.2)
	No	40 (18.8)
**Social networking site memberships, n (%)**		
	1-5	161 (75.6)
	6-10	46 (21.6)
	11-20	4 (1.9)
	≥21	2 (0.9)
**Social network sites (repeated response), n (%)**		
	KakaoTalk	210 (98.6)
	Facebook	187 (87.8)
	Instagram	170 (79.8)
	Naver Band	117 (54.9)
	KakaoStory	109 (51.2)
	Twitter	67 (31.5)
	Tumblr, Snapchat, WeChat, Flicker, Pinterest, WhatsApp, LinkedIn, and Others	68 (31.8)
**Time spent on social network sites daily, n (%)**		
	Less than 1 hour	20 (9.4)
	1-3 hours	122 (57.3)
	4-6 hours	61 (28.6)
	7-9 hours	9 (4.2)
	≥10 hours	1 (0.5)
**Experience of online course, n (%)**		
	Yes	147 (69.0)
	No	66 (31.0)

### Knowledge of Cybercivility

The average score for cybercivility knowledge was 11.30 (SD 1.86) out of a total of 15 points. The minimum and maximum scores were 5 and 15 points, respectively. Six out of the 15 items were answered correctly by 90% or more of respondents. Three items (items 1, 13, and 14) were correctly answered by 50% or less of the respondents (Table 2). Among them, item 1 received the lowest number of correct answers. Two of these items (items 1 and 14) are related to online privacy protection. The scale used to measure knowledge of cybercivility was a binary scale that assumed possible values of 0 or 1, making it inappropriate for factor analysis. The mean experience measured by frequency was 1.96 (SD 0.78) and the mean perception measured by acceptability was 1.84 (SD 0.72).

**Table 2:** Participants’ knowledge of cybercivility, experience with cyberincivility, and acceptability of cyberincivility (N=213).

Content	Correctly answered, n (%)
An organization ensures that all information it collects about users will be kept confidential.	54 (25.4)
Cyberbullying is a form of incivility that occurs in cyberspace where online communication happens.	203 (95.3)
Cyberincivility is a concern among general college populations, but it has nothing to do with students’ learning outcomes.	153 (71.8)
Cyberincivility occurs in social media channels, online learning environments, and email.	195 (91.5)
Ethical standards guiding appropriate use of social media and online networking forums in education are already well-established.	134 (62.9)
People say and do things online that they would not say or do in person.	202 (94.8)
Posting unprofessional content online can reflect unfavorably on health profession students, faculty, and institutions.	192 (90.1)
People tend to be ruder online than they are in everyday life. (Original: Americans encounter incivility almost equally offline and online)	206 (96.7)
Unlike traditional bullying, cyberbullying does not require repeated behavior.	184 (86.4)
Cyberincivility is linked to higher stress levels, lower morale, and incidences of physical harm.	199 (93.4)
Using social media inappropriately cannot lead to civil or criminal penalties.	160 (75.1)
Cyberincivility does not occur in the workplace.	184 (86.4)
Humor, anger, and other emotional components of online messages are the same as face-to-face messages.	98 (46.0)
Breaches of confidentiality on social media may lead to mandatory reporting to licensing and credentialing bodies.	113 (53.1)
Despite privacy settings on social media, nothing is private after it is posted on the internet.	129 (60.6)

The I-CVI of cybercivility knowledge was more than 0.80 and the S-CVI/Ave was 0.92. The criterion validity of the knowledge scale was tested using the known-groups technique. The knowledge of cybercivility among those who took at least one online class was 11.38 (SD 1.85) and was 11.26 (SD 1.87) for those who did not take online classes, which did not differ significantly (*t_125_*=0.437, *P*=.96). The reliability of the knowledge scale assessed by KR-20 was 0.22.

### Experience With Cyberincivility

#### Content Validity

The average score for experience with cyberincivility was 1.96 (SD 0.78) out of 5 points. The I-CVI of cybercivility experience was more than 0.80 and S-CVI/Ave was 0.98.

#### Exploratory Factor Analysis

The KMO measure of sampling adequacy for experience with cyberincivility yielded an index of 0.94. Bartlett test of sphericity was significant (Chi square=5568.63, *P*<.001), indicating that the data were appropriate for EFA. When the EFA was run using maximum-likelihood estimation with oblique promax rotation, 4 factors with eigenvalues≥1.0 were extracted and accounted for 72.22% of the overall variance. The factor analysis showed that the commonalities of all items were 0.40 or higher and the eigenvalues were 1 or greater, resulting in 4 factors being extracted. The first item was “Blaming technology for failure of communication, assignment completion, or submissions.” This was excluded from the EFA because it was not included in any factor. CFA was then performed using the 4 factors extracted in the EFA. We named these four factors F1 (individual behaviors in online environments), F2 (online class attendance attitude), F3 (email manner in online environments), and F4 (online assignment ethics).

#### Confirmatory Factor Analysis

**Figure 1:**
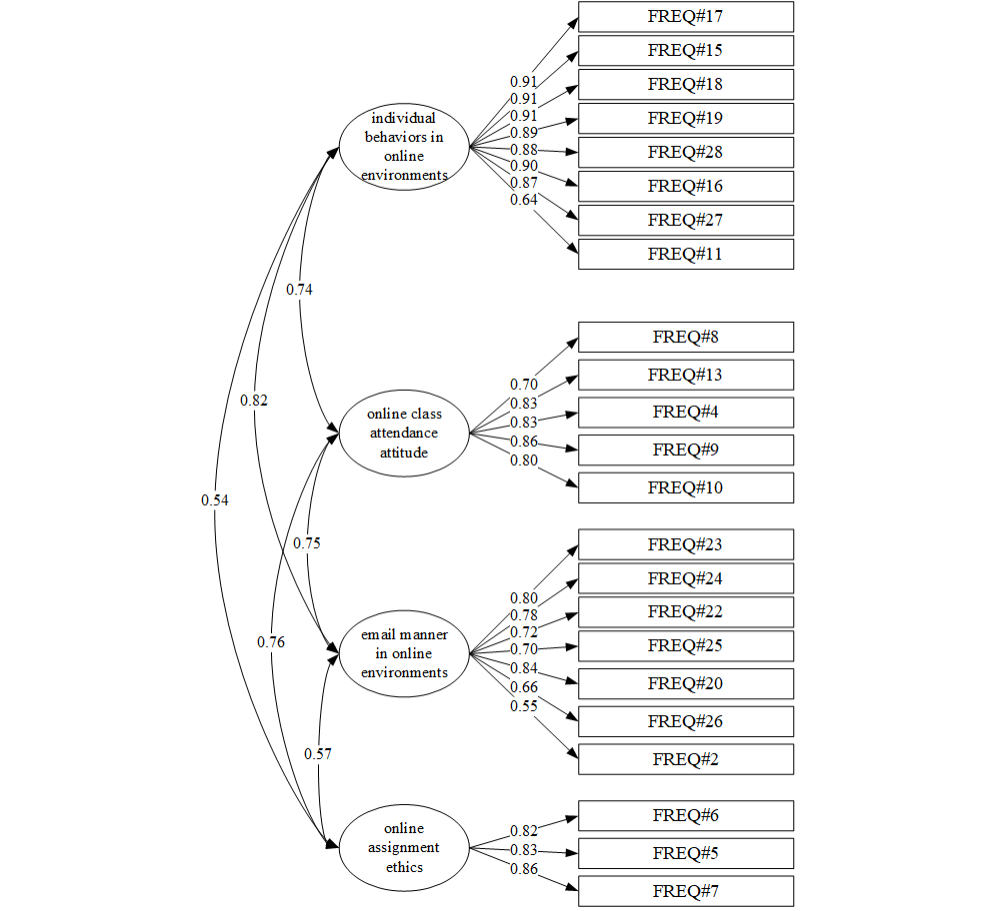
Confirmatory factor analysis of experience of cyberincivility.

A CFA was performed on the 4 subfactor models extracted via the EFA. The path models are shown in Figure 1. The goodness-of-fit indices of the 4-factor structural model were as follows: CMIN/DF=2.724, TLI=0.90, incremental fit index (IFI)=0.92, SRMR=0.06, CFI=0.92, and RMSEA=0.08. The best-fit model criteria were CMIN/df≤3, TLI≥0.90, IFI≥0.90, SRMR≤0.08, CFI≥0.90, and RMSEA≤0.08, which suggests that all of the goodness-of-fit indices of the model satisfied the criteria.

#### Reliability

Cronbach alpha coefficient of experience with cyberincivility was .96, and the Cronbach alpha coefficients of the 4 subareas were .96 for F1 (individual behaviors in online environments), .90 for F2 (online class attendance attitude), .88 for F3 (email manner in online environments), and .88 for F4 (online assignment ethics).

#### Criterion Validity

The content validity of experience was good, but the criterion validity was low. The experience of cyberincivility [14] was calculated using Pearson correlation coefficient, which was not significant (*r*=0.085, *P*=.22).

### Acceptability of Cyberincivility

#### Content Validity

The average score for acceptability of cyberincivility was 1.84 (SD 0.72) out of 5 points. Criterion validity was tested by comparing the means of correlations of the digital citizenship instrument [15].

#### Exploratory Factor Analysis

The KMO measure of sampling adequacy for acceptability of the cyberincivility items yielded an index of 0.94. Bartlett test of sphericity was significant (Chi square=5635.51, *P*<.001), indicating that the data were appropriate for EFA. When the EFA was run using maximum-likelihood estimation with oblique promax rotation, the commonalities of all items were 0.40 or higher, and the eigenvalues were 1 or greater, resulting in 4 factors being extracted. The overall explanatory power of the analysis was 70.53%, and all values conformed to the goodness-of-fit criteria.

#### Confirmatory Factor Analysis

**Figure 2:**
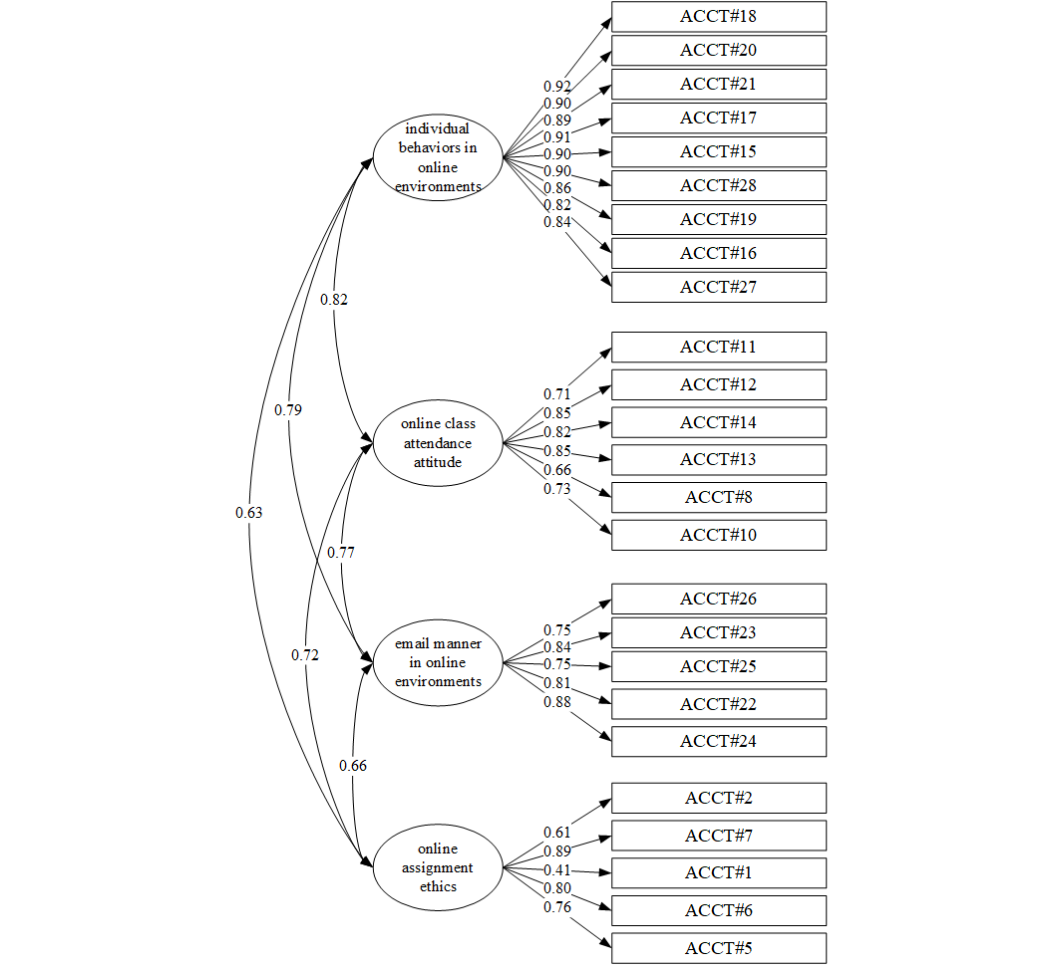
Confirmatory factor analysis of acceptability of cyberincivility.

A CFA was performed on the 4-factor model based on the 4 subfactors extracted in the EFA; the path model is presented in Figure 2. The goodness-of-fit indices of the 4-factor structural model were as follows: CMIN/df=2.343, TLI=0.92, IFI=0.93, SRMR=0.05, CFI=0.93, and RMSEA=0.08. All of the goodness-of-fit indices of the model satisfied the criteria.

#### Reliability

Cronbach alpha coefficient of the acceptability of cyberincivility was .96. Cronbach alpha coefficients of the subcategories were .97 for F1 (individual behaviors in online environments), .89 for F2 (online class attendance attitude), .90 for F3 (email manner in online environments), and .81 for F4 (online assignment ethics).

#### Criterion Validity

Criterion validity was tested by means of correlations with the digital citizenship instrument [15]. The acceptability of cyberincivility was tested using Pearson correlation coefficient, which was statistically significant (*r*=0.16, *P*<.001).

## Discussion

### Principal Findings

A Korean version of the cyberincivility assessment questionnaire was developed to test knowledge of cybercivility and to explore the experiences and acceptability of uncivil behavior in various cyber domains. The translated questionnaire was then used to measure the knowledge, experiences, and acceptability of nursing students.

Most of the participants of this study were female students who had completed 2 years toward their degrees, a nursing practicum, and at least one online lecture. Since female students make up the majority of nursing students, 80% of the participants were female. In the original study in the United States in which 205 students participated, most of them were also female at 83.4% [14]. Comparing the two studies, 75% of the participants in the current study and 82% of the US participants [14] reported that they were members of 1-5 SNSs.

The content of the Korean scale was deemed to be appropriate to measure the knowledge of cybercivility, experience, and acceptability of cyberincivility as shown in the CVI. Polit et al [21] recommended that for a scale to be judged as having excellent content validity, it should comprise items with I-CVIs of 0.78 or higher for three or more experts and an S-CVI/Ave of 0.90 or higher. The content validity using I-CVI and S-CVI/Ave of the knowledge, experiences, and acceptability scales satisfied these standards. This might reflect sufficient reviews during the translation by scholars and professors of a Korean expert in second languages.

The average score for cybercivility knowledge was similar to the result of the original study (11.30, SD 1.86 vs 11.53, SD 1.99, respectively) [14]. However, the current study’s reliability of cybercivility knowledge was lower than that observed in the original study. The low rate of correct answers may have been due to participants’ lack of knowledge or exposure to certain types of cyberactivity. The fact that traditional lectures are more common in South Korea might have been a factor contributing to this difference. In addition, faculty tend to use one-way communication in asynchronistic online classes, such as uploading lectures with voice recordings instead of holding discussions on online portals or conducting virtual online meetings with students in South Korea. Indeed, in a study that examined 2600 courses at one university in South Korea, only 3% of online courses used internet-based discussions and only 8% used online meeting programs to facilitate group work [27]. In addition, the participants of this study were undergraduate students with a mean age of 22.58 (SD 0.78) years, whereas the subjects of the original study were graduate students with a mean age of 29.70 (SD 7.0) years [14]. This age difference might have led to a difference in their experience and knowledge and caused the variation in their responses. It is recommended that future studies consider these results and reconfirm KR-20 with a greater variety of subjects.

The digital citizenship instrument [15] was used to test the criterion-related validity with the translated cyberincivility assessment instrument. The differences between the tests were likely related to their different contexts and populations. That is, the digital citizenship instrument was composed of items designed to address general social relationships on internet use or internet political participation, whereas the cyberincivility assessment questionnaire was developed to address the academic environment from the perspective of students in health-related majors [14]. However, due to differences in communication methods or meanings in different sociocultural environments [28], the definition of cybercivility can vary among individuals and countries. In the case of the United States, it is common to use email for personal and business exchanges and to share opinions in learning management systems during online classes or traditional classes with this online component [29], so that students in a US academic setting are more accustomed to these methods of communication. However, in South Korea, text messaging services using mobile phones are the most frequently used channel of communication among students and faculty [30]. Hence, it is not surprising that differences were found in what constitutes civil or uncivil behavior between these two populations. Furthermore, communication methods and manners in text messaging services are more of a concern than emails or online college communication tools [27].

The significance of the present study lies in the validity of an instrument that can measure the cybercivility knowledge and practices of South Korean nursing students whose professionalism and attitudes are important to their careers and patient care. For future study, to better understand an individual’s experience of cyberincivility, there is a need for measuring an individual’s direct experiences in addition to observations of others’ behaviors in online environments. It is also necessary to modify the knowledge cybercivility assessment questionnaire by considering cultural aspects to obtain a good reliability coefficient. The ways in which individuals communicate and their manners in cyberspace differ between South Korea and the United States, necessitating a revision of the instrument to take into account cultural differences and to provide greater validity for future studies.

### Limitations

The experience scale is limited because findings are based on self-reports of students’ personal experiences and their observations of others. Although the correlation between experience/acceptability of cyberincivility and the digital citizenship score was significant, care should be exercised in interpreting the results because the instrument might not measure exactly what it was intended to measure due to the low correlation. In addition, owing to cultural differences related to cybercivility, an English instrument rewritten in Korean might have resulted in some words getting lost in translation. The same difficulty would be true for translations into any other languages. Finally, when participants respond to a survey, they tend to give socially favorable responses, which is even more evident for Koreans than Westerners [31].

### Conclusions

This study extended and reevaluated the US version of cybercivility scales in a culturally distinct context. The results show that among the three dimensions of cybercivility (knowledge, experience, and acceptability), acceptability is well-validated while the knowledge dimension requires reexamination in the South Korean context. The content validity of experience was good, but the criterion validity was low. This study calls for more attention to contextualized cybercivility scales among health professions in countries outside the United States.

## Appendix

Multimedia Appendix 1 Korean translation of the experience and acceptability items of the Academic Cyberincivility Assessment Questionnaire.

## Abbreviations

ACAQ: Academic Cyberincivility Assessment Questionnaire
CFA: confirmatory factor analysis
CFI: comparative fit index
CMIN: minimum Chi square
CMIN/df: minimum Chi square/degrees of freedom
CVI: content validity index
EFA: exploratory factor analysis
I-CVI: content validity index for individual items
IFI: incremental fit index
IPE: interprofessional education
KMO: Kaiser-Meyer-Olkin
KR-20: Kuder-Richardson formula 20
RMSEA: root mean square error of approximation
S-CVI/Ave: content validity index for scales
SNS: social networking service
SRMR: standardized root mean square residual
TLI: Tucker-Lewis index
