# Supplementary material for: The Korean Version of the Academic Cyberincivility Assessment Questionnaire for Nursing Students in South Korea: Validity and Reliability Study
Source: J Med Internet Res. 2020 May 5;22(5):e15668. doi: 10.2196/15668 (PMC7238088; doi:10.2196/15668)
Supplement: Multimedia Appendix 1 [file jmir_v22i5e15668_app1.docx]

**Appendix 1.** Experience and acceptability items of cybercivility

|  | English version | Korean version |
| --- | --- | --- |
| 1 | Blaming technology for failure of communication, assignment completion or submissions | 의사소통에 문제가 생기거나 과제를 완료 또는 제출하지 못했을 때, 이것을 기술(테크놀러지) 탓으로 돌림 |
| 2 | Falsifying/fabricating research data | 온라인상에서 연구 자료를 변조/위조함 |
| 3 | Working on an assignment with others (via email or Instant Messaging) when the instructor asked for individual work | (이메일이나 메시지를 통해) 개인 과제를 다른 사람들과 나눠서 함 |
| 4 | Using digital technology (such as text messaging) to get unpermitted help from someone during a test or examination | 퀴즈 혹은 시험 중에 디지털 기술(예: 텍스트 메시지) 등을 사용하여 부정행위를 함 |
| 5 | Paraphrasing a few sentences of material from a written source without footnoting or referencing it in a paper | 참고자료의 출처를 밝히지 않고 온라인상에서 가져온 몇 문장을 변형하여 과제에 사용함 |
| 6 | Turning in a paper obtained in large part from a website or a term paper "mill" | 과제물 작성 시 온라인 사이트 등에서 구한 자료의 상당 부분을 복사하여 그대로 사용함 |
| 7 | Copying material from any written source and turning it in as your own work | 온라인상에 이미 나와 있는 자료 등을 복사하여 자기가 한 과제인 것처럼 제출함 |
| 8 | Not participating in required postings in discussion boards | 온라인 수업 시 필수 토론방에 참여하지 않음. |
| 9 | Not doing their part in a group activity | 온라인 수업 그룹 과제 시 그룹 활동에서 자신이 맡은 부분을 하지 않음 |
| 10 | Posting short, terse responses that do not add meaning to the online discussion | 온라인 수업 토론방에서 무의미하고 짧은 말 또는 학습에 도움이 되지 않는 말을 함 |
| 11 | Becoming offended easily by opposing ideas | 사이버 공간에서 반대 의견에 대해 쉽게 불쾌해짐 |
| 12 | Attacking (insulting, bad mouthing, cursing, rudely criticizing) other students’ thoughts or group members’ comments | 사이버 공간에서 다른 학생의 생각이나 그룹의 의견을 공격함 (모욕, 욕설, 비꼬는 말, 무례하게 비평함 등) |
| 13 | Being argumentative toward / hostilely communicating with the instructor on discussion boards or via email | 온라인 수업 토론방이나 이메일을 통해 강사와 논쟁적 또는 적대적으로 의사소통함 |
| 14 | Using displays of attitude such as capitalizing or boldfacing words in an argument | 사이버 공간의 토론에서 상대방을 조롱하는 듯한 신조어나 줄임말을 사용함 |
| 15 | Making racial, ethnic, sexual or religious insults | 온라인상에서 인종적, 민족적, 성적 또는 종교적인 모욕을 함 |
| 16 | Flooding an online environment with comments or messages | 온라인 클래스에서 상대방이나 그룹을 고려하지 않고 개인의 의견이나 메시지를 과도하게 올림 |
| 17 | Using sexually suggestive words or images | 온라인상에서 성적인 것을 암시하는 단어나 이미지를 사용함 |
| 18 | Posting or distributing an offensive picture | 온라인상에서 불쾌한 사진을 게시 혹은 배포함 |
| 19 | Misrepresenting one’s identity | 온라인상에서 개인정보를 허위 진술함 |
| 20 | Breaching student privacy | 온라인상에서 학생의 프라이버시를 침해함 |
| 21 | Breaching patient privacy | 온라인상에서 환자의 프라이버시를 침해함 |
| 22 | Not responding to emails as expected | 응답이 요구되는 이메일에 답변하지 않음 |
| 23 | Using text acronyms or abbreviations in professional emails | 전문적인 업무용 이메일에서 약어나 줄임말을 사용함 |
| 24 | Using the “reply all” button at will | 마음대로 또는 별다른 생각없이 "모두에게 답장" 버튼을 과도하게 사용함 |
| 25 | Sending time-sensitive information and expecting an immediate response | 시간을 다투는 이메일을 보낸 후 상대방의 상황을 고려하지 않고 즉각적인 반응을 기대함 |
| 26 | Sending an email without a meaningful subject | 제목란에 제목 없이 이메일을 보냄 |
| 27 | Posting derogatory remarks about one’s institution | 어떤 기관에 관한 경멸적인 발언을 온라인에 게시함 |
| 28 | Posting derogatory remarks about another profession | 다른 직업에 대한 경멸적인 발언을 온라인에 게시함 |
